# An Efficient UPLC-MS/MS Method for the Determination of Pyrroloquinoline Quinone in Rat Plasma and Its Application to a Toxicokinetic Study

**DOI:** 10.3390/molecules27227947

**Published:** 2022-11-17

**Authors:** Qingmei Sun, Yawen Hong, Zhaoxu Yang, Peixia He, Chao Chen, Jincheng Wang, Qinjie Weng

**Affiliations:** 1 Center for Drug Safety Evaluation and Research, College of Pharmaceutical Sciences, Zhejiang University, Hangzhou 310058, China; 2 Zhejiang Province Key Laboratory of Anti-Cancer Drug Research, College of Pharmaceutical Sciences, Zhejiang University, Hangzhou 310058, China

**Keywords:** PQQ disodium salt, UPLC-MS/MS, toxicokinetic, rat plasma

## Abstract

Pyrroloquinoline quinone (PQQ) is a powerful antioxidant coenzyme existing in diet, benefiting growth, development, cognition function, and the repair of damaged organs. However, a method for detecting PQQ in vivo was rarely described, limiting the research on the bioanalysis and metabolic properties of PQQ. In this study, a novel, simple, and efficient ultra-high performance liquid chromatography/tandem mass spectrometry (UPLC-MS/MS) method was developed and validated to quantify the concentration of PQQ in rat plasma. Detection through mass spectrometry was operated by multiple reaction monitoring (MRM) in negative electrospray ionization mode with ion transitions *m*/*z* 328.99→197.05 for PQQ and *m*/*z* 280.04→195.04 for the internal standard. The calibration curves were linear up to 10,000 ng/mL, with a lower limit of quantitation of 10 ng/mL. Inter-run and intra-run precision ranged from 1.79% to 10.73% and accuracy ranged from −7.73% to 7.30%. The method was successfully applied to a toxicokinetic study in Sprague–Dawley rats after the oral administration of PQQ disodium salt at doses of 250 mg/kg, 500 mg/kg, and 1000 mg/kg. The toxicokinetic parameters were subsequently analyzed, which may provide valuable references for the toxicokinetic properties and safety evaluation of PQQ.

## 1. Introduction

Pyrroloquinoline quinone (PQQ), an aromatic tricyclic o-quinone, was proven to function as a redox cycle coenzyme in bacteria and other eukaryotes [1]. The primary source of PQQ in the human body is diet, including vegetables, meat, and milk [2]. To date, PQQ was proven to play an essential role in mammals, influencing numerous physiological and biochemical processes [3,4]. A deficiency of PQQ results in growth impairment, abnormal immune responses, and reproductive dysfunction in mice [5]. Due to the versatile roles of PQQ, it is classified as a newcomer to vitamin B [6]. In addition, studies also showed the effect of PQQ in disease prevention. PQQ exhibits therapeutic effects on cognitive impairments and liver injury through multiple aspects, including ameliorating oxidative stress, inhibiting lipopolysaccharide-induced inflammation, and reducing DNA damage [7,8,9,10]. Dietary supplements containing PQQ, generally in the form of water-soluble PQQ disodium salt (PQQ Na_2_ salt), are currently approved in Canada and the United States for health maintenance [11].

As a natural health product, the safety of PQQ Na_2_ salt is noteworthy. The histopathological and hematological biochemical results for PQQ Na_2_ salt were reported [2,12] after oral administration to rats. However, toxicokinetic data for PQQ Na_2_ salt, which could quantitatively describe exposure and the relationship between internal and external doses, were never clarified [13,14]. In addition, there is limited in vivo research reporting analytical methods for PQQ Na_2_ salt. Natsumi Noji et al. developed a method combining high-performance liquid chromatography (HPLC) with electrospray-ionization (ESI) tandem mass spectrometry (MS/MS) to rapidly detect PQQ in solid and liquid food after extraction [15]. Another research report, published by Chikara Kato et al., also developed a method for quantifying PQQ in foods using enzymatic and LC-MS/MS methods [16]. Several new methods were also developed to analyze PQQ in other substrates, including specific neurotransmitter amino acid reaction mixtures and PQQ-derivative mixtures [17,18,19]. However, these methods were not validated for in vivo analysis. A gas chromatography/mass spectrometry method, established by Takeshi Kumazawa, analyzed PQQ in human and rat samples using a complicated pretreatment [20]. Mizuho Fukuda et al. optimized and validated a method for determining PQQ in human plasma via HPLC with chemiluminescence detection (CL) and solid-phase (SPE) extraction [18]. However, the extraction process was complicated and run time was up to 15 min, which is time-consuming for multiple sample analysis. Thus, a more efficient and convenient method is still required for rapid detection of PQQ concentration in plasma.

In this study, a rapid and efficient Ultra performance liquid chromatography tandem mass spectrometry (UPLC-MS/MS) method was developed to determine the concentration of PQQ Na_2_ salt in rat plasma. Using the multiple reaction monitoring (MRM) scan mode, triple quadrupole tandem mass spectrometry can achieve high selectivity and sensitivity [21]. The plasma was pre-treated using the protein precipitation method, which was simple and convenient. The linearity of this method ranged from 10 to 10,000 ng/mL and the run time was 3 min. Subsequently, this method was successfully applied to a toxicokinetic study of PQQ Na_2_ salt after oral administration to rats, providing possible perspective for explaining the in vivo toxicity of PQQ [22].

## 2. Results and Discussion

### 2.1. Development of Method

#### 2.1.1. Conditions of Mass Spectrometry

**Figure 1:**
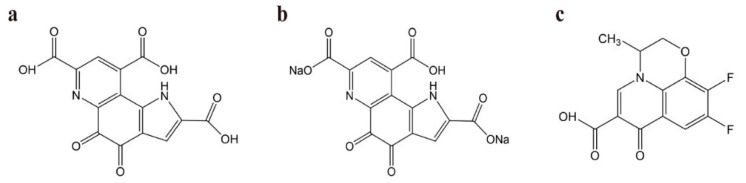
The chemical structure of PQQ (**a**), PQQ disodium salt (**b**) and IS (**c**).

In the current study, ESI (electrospray ionization) was used to generate ions because of its high sensitivity and fragmentation. Positive (ESI+) and negative (ESI−) ion-detection modes were both explored to optimize the ESI conditions for the detection of PQQ Na_2_ salt and the internal standard (IS), whose chemical structures are shown in Figure 1. The compounds showed a good response and low background signal in the negative (ESI−) mode, which was applied in the detection. Ion response is related to collision energy, cone voltage, ion source temperature, and desolvation gas flow. Thus, these parameters were optimized via manual manipulation to obtain a high response and sensitivity [23]. As shown in Table 1, the mass transition for PQQ was *m*/*z* 328.99→197.05; for IS, it was *m*/*z* 280.04→195.04. The collision energies of PQQ and IS were 26 eV and 18 eV, respectively. The cone voltages of PQQ and IS were 31 V and 34 V. The source temperature was optimized to 150 °C. The desolvation temperature was 600 °C and gas flow was 1000 L/h.

#### 2.1.2. Conditions of Liquid Chromatography

To better separate the compounds in the chromatographic column, several columns—mobile phase, flow rate, and sample injection volume—were tested in the study. Firstly, two types of columns were taken into consideration. The peak using Waters Xbridge C18 (2.1 mm × 50 mm, 3.5 µm) was wider than that using Waters ACQUITY UPLC BEH C18 (2.1 mm × 50 mm, 1.7 µm); the latter was, therefore, chosen for the experiment. The mobile phase was then tested, including water/acetonitrile, water/methanol, and water/acetonitrile–methanol (1:1, *v*/*v*). However, the methanol had little polarity and there was no peak under this condition. The water/acetonitrile mobile phase exhibited a bifurcated peak that required improving. As an ion-pairing agent, dibutylamine acetate possesses the ability to improve molecular retention. After changing the mobile phase to 10 mM dibutylamine acetate-water solution/acetonitrile, the peak shape improved significantly. Finally, the flow rate and sample injection volume were investigated at 0.5 mL/min and 5 μL; this resulted in interference by a bifurcated peak for the former and mixed peaks for the latter. Under the conditions of a 0.3 mL/min flow rate and 2 μL injection volume, the shape of the peak was excellent.

### 2.2. Method Validation

#### 2.2.1. Selectivity

**Figure 2:**
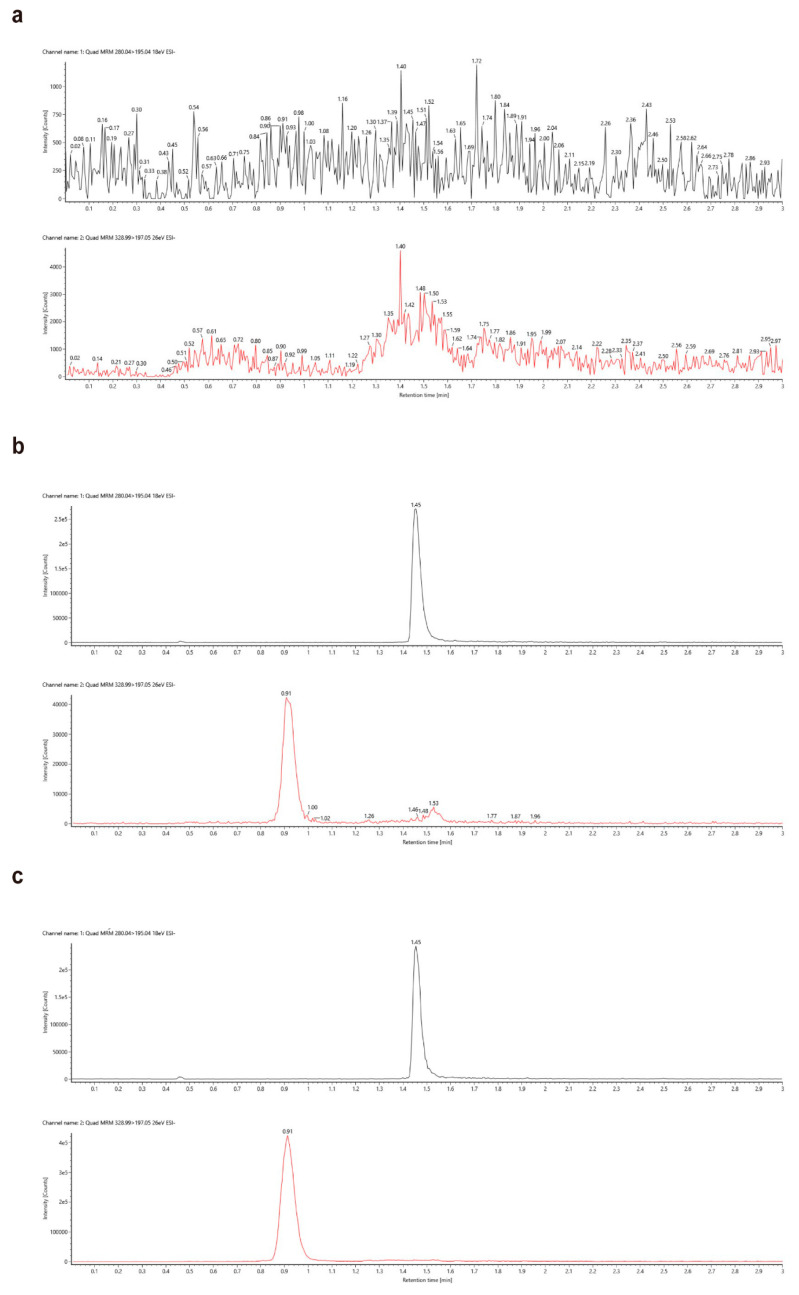
Chromatograms of IS and PQQ in rat blank plasma (**a**), spiked plasma at LLOQ (**b**) and rat plasma samples after oral administration of PQQ disodium salt (**c**).

The MRM chromatography of PQQ and IS in blank plasma, spiked plasma, and real plasma samples is shown in Figure 2. The PQQ response of double blank (DB) samples at the retention time did not exceed 20% of the LLOQ response, while the IS response of the DB samples did not exceed 5% of the LLOQ response. The results show that there was no significant peak in the retention time of PQQ and IS in the DB samples, indicating no endogenous inference in the plasma.

#### 2.2.2. Linearity and Carry-over

The linearity of the method was evaluated by analyzing the calibration curve in plasma, using least squares regression of the peak area ratios of PQQ and IS (y) versus the standard concentration of PQQ (x) with a weight factor. Linearity demonstrated a wide linear range (from 10 to 10,000 ng/mL), with an excellent correlation coefficient (r > 0.99). The LLOQ of the method was 10 ng/mL, representing the sensitivity of the method as 10 ng/mL. Carry-over samples were also evaluated in this assay, with less than 20% for PQQ and 5% for IS in each separate batch, respectively.

#### 2.2.3. Precision and Accuracy

Four concentrations of PQQ were assessed to evaluate precision and accuracy. As listed in Table 2, the intra-day and inter-day precision of the QC samples ranged from 1.79 to 10.73% and the accuracy of the QC samples ranged from −7.73 to 7.30%, which were all within 15%, indicating the method was reliable and accurate.

#### 2.2.4. Matrix Effect and Recovery

The matrix effect was assessed at LQC and HQC levels by comparing the peak area ratios of the post-extract blank plasma to the same level solution in pure solvent. From the experimental data, the mean matrix effects at LQC and HQC levels were 90.73% and 100.73%. The coefficient of variation of the matrix effect data from six batches did not exceed 15%. The results show that the different sources of the blank matrix had no effect on the analytes.

Recovery was evaluated by comparing the response of PQQ between the post-extract samples and the extracted samples at the LQC, MQC, and HQC levels. The mean recovery of QCs was from 65.26 to 73.26% and the RSD% was less than 15%, indicating the recovery was consistent. The results are shown in Table 3.

#### 2.2.5. Stability

As shown in Table 4, the stability results illustrate that PQQ was stable in plasma samples for 19.5 h at room temperature, for 106 days at −80 °C, and for three freeze–thaw cycles. It was also stable after being processed for 19.5 h at 4 °C and room temperature. The average deviation of HQC and LQC between the evaluated values and accurate values did not exceed 15%. Therefore, the method was proved to be stable throughout the experiment.

#### 2.2.6. Dilution Integrity

The results of dilution integrity show that the accuracy and precision of the samples were within 15% after a five-fold dilution, indicating that PQQ plasma samples were stable after a five-fold dilution. The results are shown in Table 5.

### 2.3. Application to Toxicokinetic Study

**Figure 3:**
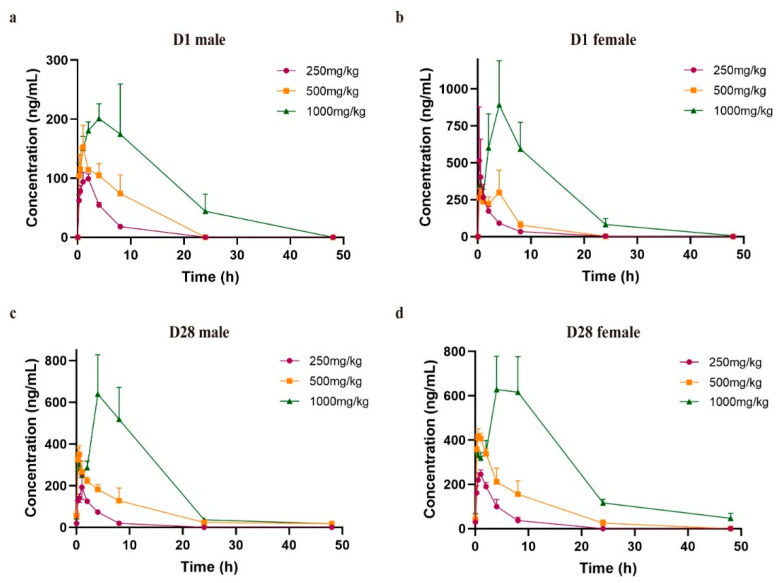
Mean plasma concentration–time profiles of SD rats after oral administration 250 mg/kg, 500 mg/kg, and 1000 mg/kg PQQ disodium salt: (**a**,**b**) plasma concentration of PQQ disodium salt in males and females on day 1; (**c**,**d**) plasma concentration of PQQ disodium salt in males and females on day 28. Each point and bar represent the means ± SEM (n = 3). Concentrations below the LLOQ (BQL) are presented as a concentration of 0 ng/mL to maintain the integrity of the curves and are excluded from statistics of pharmacokinetic parameters.

After a single oral administration of PQQ Na_2_ salt to rats, the developed and validated method was applied to the toxicokinetic experiment. The plasma–concentration of PQQ Na_2_ salt varies with time: the calculated toxicokinetic parameters are shown in Figure 3 and Table 6. The results show that PQQ Na_2_ salt was rapidly absorbed after oral administration, the average T_max_ for doses of 250 mg/kg, 500 mg/kg, and 1000 mg/kg on day 1 being 1.50 ± 0.55 h, 1.29 ± 1.36 h, and 4.83 ± 2.71 h, respectively. Rapid absorption was also observed for repeat doses of PQQ Na_2_ salt to rats on day 28, with a T_max_ of 1.00 ± 0.00 h, 1.21 ± 1.40 h, and 4.67 ± 1.63 h, respectively. The average T_1/2_ for different doses of PQQ Na_2_ salt was 2.18 ± 0.32 h, 3.26 ± 1.01 h, and 10.23 ± 8.87 h on day 1, and 2.58 ± 1.19 h, 7.46 ± 5.94 h, and 8.74 ± 2.70 h on day 28, indicating that the elimination of PQQ Na_2_ salt was slow, especially in the high-dose group. In addition, with the increasing dose of PQQ Na_2_ salt, both the AUC_(0-t)_ and C_max_ increased in all groups, providing a reference for the dose–exposure relationship. Twenty-four hours after administration, PQQ Na_2_ salt was rarely detected in the rat plasma in the low- and medium-dose groups, whereas it could still be detected in the high-dose group; it was, however, completely eliminated forty-eight hours after administration. These results suggest that high-dose administration of PQQ Na_2_ salt may have a longer retention time in the body compared with that of low- and medium-dose administration.

The relations between toxicokinetic parameters and other factors, including single or repeat dosing and gender, were also considered. From the plasma concentration data for D28 at pre-dose, PQQ can be detected at dozens of ng/mL, demonstrating it was not completely eliminated following the previous administration. After repeated intragastric administration for four weeks, the AUC_(0-t) D28/D1_ for different doses was 1.38, 2.88, and 2.06, respectively, and the C_max D28/D1_ for different doses was 1.48, 1.71 and 1.98, indicating that the systemic exposure to PQQ in rats on D28 was a little higher than that on D1. Thus, our results indicate that there might be a slight accumulation of PQQ Na_2_ salt in rats following repeated administration for 28 days, leading to the different systemic exposure to PQQ between a single dose and 28 days’ administration. Comparing the parameters in male and female rats, the C_max_ for females was more than twice as high as the C_max_ for males on day 1. However, on day 28, the C_max_ for females/males was 1.03, 1.00, and 0.78 for the different doses, respectively; the same results were also observed for the AUC_(0-t)_. These results indicate that the exposure to PQQ Na_2_ salt for males and females on day 1 might be different, and it may be necessary to design rational dosage regimens for men and women.

## 3. Materials and Methods

### 3.1. Chemicals and Reagents

Standard PQQ (98.0%) was obtained from Dalian Meilun Biotechnology Co., Ltd. (Dalian, China). IS reference Levofloxacin Impurity 27 (94.37%) was obtained from SINCO PHARMACHEM Inc. (Middletown, DE, USA). The structures of PQQ and IS are shown in Figure 1. HPLC grade methanol and acetonitrile were purchased from Merck KGaA (Darmstadt, Germany). Dibutylamine acetate was purchased from Tokyo Chemical Industry Co., Ltd. (Tokyo, Japan). Ammonium hydroxide was purchased from Aladdin Reagent Co., Ltd. (Shanghai, China). Purified water was prepared with a Milli-Q water purification system from Merck KGaA (Darmstadt, Germany).

### 3.2. Instruments

The assay was performed with a triple-quadrupole ultra-high performance liquid chromatography (UPLC) system (Xevo TQ-S, Waters Corp., Milford, MA, USA). Unifi 1.9.3 software (Waters Corp., Milford, MA, USA) was used to collect the analyzed data. The chromatographic separation of the samples was performed on an ACQUITY UPLC BEH C18 column (2.1 mm × 50 mm, 1.7 μm, Waters, Milford, MA, USA). In addition, electronic scales XS205 (METTLER TOLEDO, Greifensee, Switzerland) were used for weighing the powder. An electric separator (Multipette E3), pipettes (Research), and the centrifuge (5417R) were obtained from Eppendorf (Hamburg, Germany). A vortex mixer (Vortex 2, IKA, Staufen, Germany) was also used in the assay.

### 3.3. LC-MS/MS Conditions

As described above, a Waters ACQUITY UPLC BEH C18 (2.1 mm × 50 mm, 1.7 μm) chromatography column was used in this assay. The temperature of the column was maintained at 40 °C and the autosampler was set at 4 °C. The mobile phase was 10 nM dibutylamine acetate–water solution (DBAA, A) and acetonitrile (B). The run time was 3 min and the sample was eluted by gradient elution as follows: 0–0.40 min, 20%B; 0.40–1.00 min, 20–70%B; 1.00–2.50 min, 70%B; 2.50–2.51 min, 70–20%B; 2.51–3.00 min, 20%B. The flow rate was 0.3 mL/min. The injection volume was 2 μL.

Mass spectrometric analysis was carried out in negative ion mode with electrospray ionization. Multiple reactions monitoring (MRM) was utilized in the study and the parameters were optimized as summarized in Table 1. Mass transition for PQQ was *m*/*z* 328.99→197.05 and IS was *m*/*z* 280.04→195.04. The source temperature was 150 °C. The desolvation temperature was 600 °C and gas flow was 1000 L/h.

### 3.4. Preparation of Calibration Standard and Quality Control Solution

Two copies of stock solution of PQQ were dissolved in methanol to 1 mg/mL. The IS stock solution was first prepared in ammonium hydroxide and then dissolved in methanol. One PQQ stock solution was diluted with methanol–water (1:1, *v*/*v*) solution to a series of standard working solutions of different concentrations (0.20, 0.40, 2.0, 6.0, 20, 50, 100, 200 μg/mL). Four levels of quality control working solution (0.20, 0.60, 10, 160 μg/mL) were prepared with the other PQQ stock solution. The IS stock solution was also diluted with the same diluent to 50 ng/mL. All the stock and working solutions were stored at 4 °C.

### 3.5. Sample Preparation

A method of protein precipitation was used in this study to extract the compound from the plasma. An amount of 5 μL standard or quality control working solution was mixed with 95 μL rat plasma as the plasma sample. The concentrations of standard samples were 10, 20, 100, 300, 1000, 2500, 5000, 10,000 ng/mL and quality control samples at low, medium, high were 30, 500, 8000 ng/mL, respectively. IS stock solution was diluted to 500 ng/mL with 50% methanol–water (1:1, *v*/*v*) as the IS solution. Quantities of 50 μL IS solution and 100μL acetonitrile were added to a 50 μL plasma sample and vortexed at 1200 rpm for 5 min. Double blank (DB) samples did not contain PQQ or IS; these were prepared with a 50 μL blank plasma sample, 50μL 50% methanol–water (1:1, *v*/*v*), and 100 μL acetonitrile. Blank samples only contained IS without PQQ and were prepared with a 50μL blank plasma sample, 50 μL IS, and 100 μL acetonitrile. The mixture was centrifuged at 13,000 rpm for 5 min. Next, 50 μL supernatant and 50μL mobile phase A (10 mM DBAA) were added to plates. The samples were injected for LC-MS/MS analysis after mixing.

### 3.6. Method Validation

The validation in this study included selectivity, linearity and carry-over, precision and accuracy, matrix effect, recovery, stability, and dilution integrity.

#### 3.6.1. Selectivity

Blank plasma from six different individual sources and one blank hemolysis plasma (plasma:hemolysis blood = 100:2, *v*/*v*) were prepared as DBs and lower limit of quantification (LLOQ) samples. The samples were treated as described above (3.5 Sample preparation) and then injected for analysis. The response to PQQ and IS of the blank samples were compared to that of the LLOQ samples. Correspondence analysis was performed for each source sample.

#### 3.6.2. Linearity and Carry-Over

The linear calibration curve was established by plotting the peak area ratio of PQQ to IS and the standard concentration (from 10 to 10,000 ng/mL) of PQQ, with weighted least squares (W = 1/X^2^) to calculate the linear regression. The LLOQ of the calibration curve was determined on a signal to noise ratio of at least 10:1. The acceptable criterion was a correlation coefficient of the calibration curve of no less than 0.99 (r ≥ 0.99). The accuracy of the LLOQ was within 20% of the labeled value, and other levels of concentration were within 15% of the labeled value.

Carry-over samples were DB samples after the injection of the ULOQ samples, normally following the ULOQ in the calibration curve. It was acceptable that the peak area of PQQ in the DBs did not exceed 20% of that in the LLOQ. The peak area of IS in the DBs also did not exceed 5% of that in the LLOQ.

#### 3.6.3. Precision and Accuracy

The inter- and intra-day precision and accuracy were investigated by analyzing six LLOQ samples, six LQC samples, six MQC samples, and six HQC samples, in three separated batches, on at least two days. Precision was expressed as relative standard deviation (RSD%) and accuracy was expressed as relative error (RE%).

#### 3.6.4. Matrix Effect and Recovery

The matrix effect was evaluated by comparing the peak areas of the post-extract blank samples with those of the pure solvent. The blank plasma from six different individual sources and one hemolysis unit (plasma:hemolysis blood = 100:2, *v*/*v*) was tested with LQC and HQC samples, with three parallel samples at each concentration.

Recovery was prepared by analyzing the differences in peak areas between extracted QC samples and post-extraction spiked samples of LQC, MQC, and HQC.

#### 3.6.5. Stability

The stability of PQQ in plasma exposed to different conditions was examined. LQC and HQC samples were tested under the following conditions: plasma-sample short-term storage at room temperature; processed samples at room temperature for 19.5 h; processed samples at 2–8 °C for 19.5 h; plasma-sample long-term storage at −80 °C for 106 days; and three freeze–thaw cycles from −80 °C to room temperature. The stability of samples was evaluated by RE% between estimated and exact values.

#### 3.6.6. Dilution Integrity

The concentration of PQQ in plasma may be above the ULOQ of the calibration curve. Therefore, the samples with concentrations higher than ULOQ were diluted to be within the range of the calibration curve. An amount of 800 μg/mL working solution of PQQ was first diluted to 40 μg/mL with rat plasma. Next, 20 μL of the above samples was added to 80 μL blank plasma to obtain five-fold diluted samples of PQQ with a concentration of 8000 ng/mL. The final samples were detected after pretreatment.

### 3.7. Animals

The animal studies were approved by Zhejiang University Animal Research Committee (Approval number IACUC-20-201), and the experiments were conducted in line with the institutional guidelines. Sprague–Dawley rats (200–220 g, 6–8 weeks, 24 males and 24 females) were purchased from Vital River Laboratory Animal Technology Co. (Beijing, China). The rats were kept at a temperature of 20–26 °C and humidity of 40–70% with a 12 h light/dark cycle. All the animals were acclimated for 7 days; they fasted for 12 h and had free access to water before the experiments were performed [24].

### 3.8. Toxicokinetic Study

According to the ICH guidelines for dose settings and the results of the preliminary experiment, no dose-related toxicity was observed at 250 mg/kg; feces were green when the dose ≥500 mg/kg; and kidney weight in females increased at a dose of 1000 mg/kg. The maximum tolerated dose (MTD) of repeated administration of PQQ Na_2_ salt was greater than 1000 mg/kg. Therefore, 250 mg/kg, 500 mg/kg, and 1000 mg/kg were set as the low, medium, and high doses, respectively. Forty-eight rats were randomly assigned into four groups, each group having an equal number of males and females. The four groups were set as the control, the PQQ low-dose group (250 mg/kg), the PQQ medium-dose group (500 mg/kg), and the PQQ high-dose group (1000 mg/kg), respectively. PQQ Na_2_ salt was dissolved in normal saline. Half of the rats in the four groups (3 males and 3 females in each group) were administered a single dose of different concentrations of PQQ Na_2_ salt, as above. The day of the first administration was named day 1. Blood samples were then collected into heparinized tubes using the tail-bleed method before administration and, thereafter, at 0.25 h, 0.5 h, 1 h, 2 h, 4 h, 8 h, 24 h, and 48 h. Each time, 0.25 mL blood was collected. The remaining rats were administered with repeated dosing for four weeks and their blood samples were also taken on day 28 at pre-dose, 0.25 h, 0.5 h, 1 h, 2 h, 4 h, 8 h, 24 h, and 48 h. In addition, blood samples of rats in the control group were collected at two time points only—pre-dose and 2 h after administration. The blood samples were then immediately centrifuged at 3000 rpm for 10 min. The supernatant plasma was collected and then stored at −80 °C until analysis.

### 3.9. Data Analysis

The UPLC-MS/MS data were collected and processed with Unifi 1.9.3 and then analyzed with Microsoft Excel 2007 (Microsoft Co., Redmond, Washington, DC, USA). The toxicokinetic parameters were calculated using DAS 3.0 software (Mathematical Pharmacology Professional Committee of China, Shanghai, China). SPSS software (IBM, Chicago, IL, USA) was also used in the study to analyze the kinetic parameters.

## 4. Conclusions

In this study, a rapid and simple UPLC-MS/MS method was developed and applied in toxicokinetic experiments with PQQ Na_2_ salt in rat plasma. The linear range of the method was from 10 to 10,000 ng/mL and the method validation proved that the method is accurate, precise, stable, and reliable. After the successful application of this method in toxicokinetic studies in rats, we showed that PQQ was rapidly absorbed and slowly eliminated, especially in high-dose groups. The method and toxicokinetic data may be further applied to the in vivo detection and safety evaluation of PQQ, to provide data support and an analytical method for the development of PQQ.

## Figures and Tables

**Table 1:** Multiple reaction monitoring (MRM) parameters for analytes included in this assay.

Compound	Precursor Ion (*m*/*z*)	Product Ion (*m*/*z*)	Capillary Voltage (kV)	Collision Energy (eV)	Cone Voltage (V)
PQQ	328.99	197.05	3.00	26	31
Levofloxacin impurity 27 (IS)	280.04	195.04	3.00	18	34

**Table 2:** Inter- and intra-run accuracy and precision of the method.

Concentration of PQQ (ng/mL)	Intra-Day			Inter-Day		
	Mean ± SD	Precision (RSD%)	Accuracy (RE%)	Mean ± SD	Precision (RSD%)	Accuracy (RE%)
10	10.50 ± 1.13	10.73	5.02	10.73 ± 0.32	2.98	7.30
30	30.34 ± 2.03	6.69	1.13	28.33 ± 2.30	8.13	−5.57
500	468.40 ± 10.43	2.23	−6.32	461.33 ± 11.55	2.50	−7.73
8000	7972.00 ± 225.54	2.83	−0.35	8079.33 ± 144.78	1.79	0.99

**Table 3:** Recovery and Matrix effect of the method.

Concentration of PQQ (ng/mL)	Recovery		Matrix Effect (%)	
	Average (%)	RSD%	Average (%)	RSD%
30	65.26 ± 3.36	5.14	90.73 ± 3.72	4.10
500	72.47 ± 3.08	4.24	/	/
8000	73.26 ± 1.83	2.50	100.73 ± 1.38	1.37

**Table 4:** Stability of PQQ in rat plasma.

	Concentration of PQQ (ng/mL)	Calculated Concentration (ng/mL)	RSD%	RE%
Processed samples at 2–8 °C 19.5 h	30	28.80 ± 0.60	2.08	−4.00
	8000	9053 ± 638.46	7.05	13.17
Processed samples at room temperature 19.5 h	30	27.60 ± 0.72	2.61	−8.00
	8000	8427 ± 161.66	1.92	5.33
Plasma samples at room temperature 19.5 h	30	32.23 ± 1.05	3.26	7.44
	8000	8050 ± 141.07	1.75	0.63
Plasma samples at −80 °C 106 days	30	30.33 ± 2.59	8.54	1.11
	8000	8810 ± 316.07	3.59	10.13
Three freeze—thaw cycles (−80 °C)	30	28.87 ± 4.19	14.51	−3.78
	8000	8707 ± 156.31	1.80	8.83

**Table 5:** Dilution integrity of PQQ in rat plasma.

Concentration of PQQ (ng/mL)	Calculated Concentration(ng/mL)	RSD%	RE%
8000	8146 ± 193.98	2.38	1.83

**Table 6:** Toxicokinetic parameters of PQQ after single and 28-day repeated administration in rats at 250 mg/kg, 500 mg/kg and 1000 mg/kg dose level.

	Dose	Sex	C_max_	T_max_	t_1/2_	AUC_(0-t)_	V_z_/F	CL_z_/F	MRT
	mg/kg	Male/Female	μg/L	h	h	μg/L×h	L/kg	L/h/kg	h
D1	250	Male	111.67 ± 7.64	1.67 ± 0.58	2.44 ± 0.19	464.81 ± 24.04	1667.14 ± 180.20	473.47 ± 16.44	2.87 ± 0.11
		Female	209.00 ± 13.45	1.33 ± 0.58	1.92 ± 0.15	759.68 ± 199.68	889.45 ± 220.83	324.83 ± 99.25	2.51 ± 0.31
		Average	160.33 ± 54.20	1.50 ± 0.55	2.18 ± 0.32	612.24 ± 205.58	1278.29 ± 462.53	399.15 ± 103.32	2.69 ± 0.28
	500	Male	169.67 ± 44.47	2.00 ± 1.73	3.22 ± 0.78	818.35 ± 140.65	2444.08 ± 727.56	519.94 ± 47.08	3.56 ± 0.23
		Female	353.67 ± 114.69	0.58 ± 0.38	3.29 ± 1.40	1393.19 ± 433.09	1405.49 ± 212.89	331.96 ± 140.57	3.20 ± 0.36
		Average	261.67 ± 127.32	1.29 ± 1.36	3.26 ± 1.01	1105.77 ± 426.70	1924.78 ± 743.96	425.95 ± 139.25	3.38 ± 0.33
	1000	Male	226.33 ± 82.85	4.33 ± 3.51	13.20 ± 12.85	3157.43 ± 1335.85	3728.86 ± 2542.34	236.44 ± 89.85	8.72 ± 3.02
		Female	723.00 ± 480.63	5.33 ± 2.31	7.26 ± 2.25	7266.55 ± 4018.11	1621.20 ± 987.94	176.56 ± 143.33	7.64 ± 1.12
		Average	474.67 ± 411.28	4.83 ± 2.71	10.23 ± 8.87	5211.99 ± 3498.19	2675.03 ± 2075.69	206.50 ± 111.90	8.18 ± 2.12
D28	250	Male	234.00 ± 166.14	1.00 ± 0.00	2.50 ± 0.62	707.05 ± 126.78	1171.60 ± 369.75	323.76 ± 45.49	2.64 ± 0.45
		Female	241.67 ± 29.74	1.00 ± 0.00	2.66 ± 1.77	985.88 ± 476.52	799.02 ± 103.99	266.25 ± 138.29	2.70 ± 0.64
		Average	237.83 ± 106.83	1.00 ± 0.00	2.58 ± 1.19	846.47 ± 347.25	985.31 ± 317.27	295.01 ± 97.31	2.67 ± 0.49
	500	Male	448.67 ± 54.64	0.58 ± 0.38	10.61 ± 7.41	2552.09 ± 277.59	2678.86 ± 1440.52	188.76 ± 33.14	11.22 ± 2.14
		Female	447.00 ± 49.87	1.83 ± 1.89	4.31 ± 1.88	3810.28 ± 2001.67	820.75 ± 209.34	156.69 ± 101.20	5.21 ± 2.36
		Average	447.83 ± 46.79	1.21 ± 1.40	7.46 ± 5.94	3181.18 ± 1452.03	1749.81 ± 1372.35	172.73 ± 69.60	8.22 ± 3.86
	1000	Male	1053.00 ± 240.58	5.33 ± 2.31	7.39 ± 2.73	11,444.24 ± 2381.41	903.29 ± 210.51	88.76 ± 17.45	8.81 ± 0.21
		Female	823.33 ± 250.10	4.00 ± 0.00	10.09 ± 2.29	10,055.09 ± 2936.18	1360.58 ± 181.32	97.32 ± 30.34	10.13 ± 2.48
		Average	938.17 ± 252.97	4.67 ± 1.63	8.74 ± 2.70	10,749.67 ± 2509.15	1131.94 ± 305.96	93.04 ± 22.63	9.47 ± 1.73
D28/D21	250	Average	1.48	/	/	1.38	/	/	/
	500	Average	1.71	/	/	2.88	/	/	/
	1000	Average	1.98	/	/	2.06	/	/	/
D1	250	Female/male	1.87	/	/	1.63	/	/	/
	500		2.08	/	/	1.70	/	/	/
	1000		3.19	/	/	2.30	/	/	/
D28	250	Female/male	1.03	/	/	1.39	/	/	/
	500		1.00	/	/	1.49	/	/	/
	1000		0.78	/	/	0.88	/	/	/

## Data Availability

The data presented in this study are available on request from the corresponding author.
